# Strategies for Structural Genomics Target Selection

**DOI:** 10.1100/tsw.2002.33

**Published:** 2002-03-05

**Authors:** Teri Gaasterland

**Affiliations:** Laboratory of Computational Genomics, Rockefeller University, 1230 York Ave., New York, NY 10021, USA

## Abstract

We have developed a protein sequence analysis pipeline that ranks proteins as targets for high throughput structure determination. The ranking is designed to maximize both the biological and informational impact of new 3D protein structures solved through the structural genomics initiative. The analysis system accepts proteins from multiple genomes as input, builds sequence families based on remote homology, identifies families with one or more solved structures, and ranks the remaining families according to criteria designed to maximize structure determination efficacy, increase the likelihood of a novel fold, and maximize the number of new protein structure models that can be built from a solved structure.

